# Assessing the toxicity after moderately hypofractionated prostate and whole pelvis radiotherapy compared to conventional fractionation

**DOI:** 10.1007/s00066-023-02104-7

**Published:** 2023-06-21

**Authors:** Matthias Moll, Gregor Goldner

**Affiliations:** Department of Radiation Oncology, Comprehensive Cancer Center, Medical University of Vienna, Währinger Gürtel 18–20, A-1090 Vienna, Austria

**Keywords:** Prostate cancer, Primary treatment, Moderate Hypofractionation, Toxicity, Whole pelvis

## Abstract

**Objective:**

To evaluate acute and late gastrointestinal (GI) and genitourinary (GU) toxicities after moderately hypofractionated (HF) or conventionally fractionated (CF) primary whole-pelvis radiotherapy (WPRT).

**Methods:**

Primary prostate-cancer patients treated between 2009 and 2021 with either 60 Gy at 3 Gy/fraction to the prostate and 46 Gy at 2.3 Gy/fraction to the whole pelvis (HF), or 78 Gy at 2 Gy/fraction to the prostate and 50/50.4 Gy at 1.8–2 Gy/fraction to the whole pelvis (CF). Acute and late GI and GU toxicities were retrospectively assessed.

**Results:**

106 patients received HF and 157 received CF, with a median follow-up of 12 and 57 months. Acute GI toxicity rates in the HF and CF groups were, respectively, grade 2: 46.7% vs. 37.6%, and grade 3: 0% vs. 1.3%, with no significant difference (*p* = 0.71). Acute GU toxicity rates were, respectively, grade 2: 20.0% vs. 31.8%, and grade 3: 2.9% vs. 0%, (*p* = 0.04). We compared prevalence of late GI and GU toxicities between groups after 3, 12, and 24 months and did not find any significant differences (respectively, *p* = 0.59, 0.22, and 0.71 for GI toxicity; *p* = 0.39, 0.58, and 0.90 for GU toxicity).

**Conclusion:**

Moderate HF WPRT was well tolerated during the first 2 years. Randomized trials are needed to confirm these findings.

**Supplementary Information:**

The online version of this article (10.1007/s00066-023-02104-7) contains supplementary material, which is available to authorized users.

## Introduction

Primary irradiation for treating prostate cancer is evolving in two directions. On one hand, there is a pattern of dose escalation. Initiated in the 1990s, several studies showed that increasing the total dose used in radiotherapy for primary prostate cancer improved oncological control [1–3]. Currently, this topic is under investigation; for example, simultaneously integrated boosts are being tested for even greater dose escalations, as shown in the FLAME trial [4]. However, no androgen deprivation therapy (ADT) was used in this trial. There is evidence that dose escalation leads to omission of ADT, in both trials [5] and clinical routine [6]. Yet, for patients with high-risk prostate cancer, as of today, ADT of at least 18 months is recommended [7], as a reduction to 6 months proved to be inferior [8]. On the other hand, the importance of hypofractionation is rising; for instance promising results were shown in the CHHiP trial [9]. Hypofractionation provides several advantages, for patients, radiotherapists, and society alike. It provides greater treatment capacity, shortened treatment courses, and reduced treatment costs [10].

The goal of this study was to investigate hypofractionation in the setting of whole pelvis radiotherapy (WPRT). Although several randomized controlled trials have tested the benefit of WPRT [11–13], no definite evidence of a benefit was found. More recently, Murthy et al. were able to show a benefit for tumor control with the exception of overall survival [14]. Nevertheless, both the National Comprehensive Cancer Network guidelines (NCCN) [15] and the British National Institute for Clinical Excellence guidelines [16] recommend the use of WPRT in patients with an increased risk of lymph node involvement, particularly in patients with unfavorable intermediate-risk and high-risk prostate cancer. Combining the advantages in tumor control after WPRT with the advantages of hypofractionation is a reasonable step. However, the ASTRO guidelines on hypofractionation recommend hypofractionation only when not including the pelvic lymph nodes [17]. The same applies to the NRG guidelines, emphasizing the unestablished nature of hypofractionationed WPRT [18]. Published data in form of a meta-analysis regarding moderate hypofractionation, also including WPRT are of a more recent date [19]. Within this meta-analysis, there are only 2 studies with actual hypofractionation to the pelvis [20, 21], so evidence still remains sparse.

In the present preliminary study, we investigated treatment with moderately hypofractionated WPRT for prostate cancer, performed with intensity modulated radiotherapy (IMRT) and volumetric intensity modulated arc therapy (VMAT) techniques.

## Materials and methods

This retrospective study protocol was approved by the local ethics review board, according to local laws and regulations in accordance with the declaration of Helsinki.

All patients treated in our department between January 2009 and July 2021 were included, when they met the following criteria:

- received primary prostate cancer treatment
- underwent external beam radiotherapy (EBRT) with 60 Gy to the prostate (3 Gy single doses) and 46 Gy (2.3 Gy single doses) to the pelvic lymph nodes, or 78 Gy (2 Gy single doses) to the prostate and 50–50.4 (1.8–2 Gy single doses) to the pelvic lymph nodes
- tumors were staged as any cT, cN0/X, or cM0/X, using either CT, MRI or PET-CT
- ≥ 15% risk of positive pelvic lymph nodes, according to the Roach formula [22]

Up until 2018, patients were treated with 78/50 Gy, performed with either IMRT, with the step and shoot-technique, or with the VMAT technique. Starting in 2019, all patients were treated with 60/46 Gy, performed with the VMAT technique. The clinical target volume (CTV) was defined as the prostate and seminal vesicles, for 60 or 78 Gy delivered at 3 or 2 Gy single doses, respectively. The CTV for pelvic lymph node irradiation included the external, internal, and common iliac lymph nodes, up to the aortic bifurcation (usually L4/5). The prescribed doses were 46 Gy with 2.3 Gy single doses, for patients that received hypofractionated delivery (HF group); or 50–50.4 Gy with 1.8–2 Gy single doses for patients that received the conventional fractionation delivery (CF group). Assuming an α/β-value of 3 Gy, the single doses of 3 Gy would provide a total of 72 Gy equivalent dose (EQD_2_ _Gy_), and the single doses of 2.3 Gy would provide a total of 48.8 Gy EQD_2_ _Gy_. If the α/β-value was assumed to be 1.5 Gy, the EQD_2_ _Gy_ values would be 77.1 and 49.9 Gy, respectively. Doses were prescribed to 95% of the planning target volume (PTV), according to International Commission on Radiation Units and Measurements report 83 [23]. Dose constraints for both schedules are shown in Appendix 1. Up until the start of the COVID pandemic in March 2020 which limited capacities of our intervention rooms, all patients received gold fiducial markers (GFM), unless deemed unsafe for anesthesia. Safety margins were 5 mm for patients with implanted GFM, and 7 mm for patients without in the HF group and 7 mm for patient with GFM and 10 mm for patients without. Planning for all patients was performed using a CT scan and an MRI. All patients received a rectal balloon to immobilize the prostate [24] and were treated in the supine position with a full bladder. For the CF group, Cone-beam CT control scans were performed daily for the first week, followed by daily ExacTrac (Brainlab, Munich, Germany) controls for the rest of treatment. The HF group received daily ExacTrac controls.

ADT was prescribed at the discretion of the attending urologist, but it was recommended for 6 months, in patients with intermediate-risk prostate cancer, and for 3 years, in patients with high-risk prostate cancer [25].

Gastrointestinal (GI) and genitourinary (GU) toxicities were assessed, according to Radiation Therapy Oncology Group (RTOG) grading [26]. Toxicities were graded by the treating physician at the end of treatment, at 3 and 12 months after treatment, and once every year thereafter. Patients were always given the option to see a physician, during and after treatment, in case of side effects. Acute toxicity was defined as an adverse effect experienced during or at the end of treatment. Late toxicity was defined as an adverse effect experienced during the follow-up period.

Statistical analyses were performed with GraphPad Prism 9.3 (GraphPad Software, San Diego, USA). All statistical tests were two-sided, and *p*-values < 0.05 were considered statistically significant. Maximum toxicities were compared with the Mann-Whitney U‑Test. To analyze biochemical control we used the Kaplan-Meier method.

## Results

Patient characteristics are displayed in Table 1. Notable differences between treatment groups were ADT prescriptions, and the median follow-up times: 12 months after HF and 57 months after CF. 16 patients (15%) of the HF group and 109 (69%) of the CF group were treated with GFM, respectively.

**Table 1:** Patient characteristics

	CF	%	HF	%
*n*	157	–	106	–
*cT category*				
*1*	59	37.6	62	58.5
*2*	75	47.8	32	30.2
*3*	22	14.0	12	11.3
*4*	1	0.6	0	0.0
*iPSA in µg/l, Median (25%/75% IQR)*	12.3 (9.0/22.2)	–	12.0 (8.7/18.1)	–
*Gleason Score*				
*6 or below 6*	16	10.2	4	3.8
*7a*	41	26.1	29	27.4
*7b*	21	13.4	19	17.9
*8–10*	78	49.7	54	50.9
*X*	1	0.6	0	0.0
*NCCN risk group*				
*Intermediate*	43	27.4	40	37.7
*High*	114	72.6	64	62.3
*ADT prescribed*	128	81.5	66	62.3
*Median duration in months (25%/75% IQR)*	15 (6/30)	–	8.5 (6/15)	–
*Median age at RT (25%/75% IQR)*	74.3 (70.7/76.8)	–	77.1 (75.0/80.1)	–
*Median follow-up in months (25%/75% IQR)*	57 (24/80.5)	–	12 (3/12)	–
*RT Technique*				
*IMRT*	73	46.5	0	0.0
*VMAT*	84	53.5	106	100.0

*iPSA* initial PSA, *ADT* Androgen deprivation therapy, *EBRT* External beam radiotherapy, *IMRT* intensity modulated radiation therapy, *VMAT* Volumetric modulated arc therapy

Around half of the CF group was treated with the IMRT technique, and the other half was treated with the VMAT technique. Therefore, we performed an internal analysis to compare these modalities. We compared the maximum acute and maximum late GI and GU toxicities and the GI and GU toxicities after 3, 12, and 24 months. With the exception of the 3‑month GI toxicity, we were unable to detect any significant differences between these techniques. Most of the difference was a 10% difference in the rates of grades 0 and 1 toxicities; therefore, we decided to merge the two treatment modalities in subsequent analyses.

**Fig. 1:**
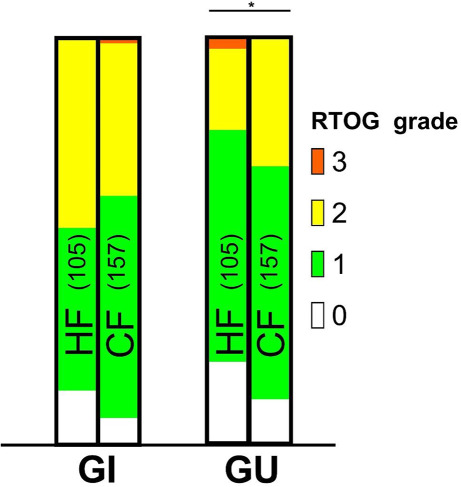
Maximum acute gastrointestinal (*GI*) and genitourinary (*GU*) toxicity. *P* = 0.71 and 0.04 for gastrointestinal and genitourinary toxicity, respectively. *HF* hypofractionation, *CF* conventional fractionation

The maximum acute toxicities are shown in Fig. 1. Less than half of the patients in both groups developed ≥ grade 2 GI or GU toxicities.

For late maximum toxicities, we detected grade 2 or higher GI toxicity in 7% of the HF group and 15% of the CF group. Grade 2 or higher GU toxicity was detected in 11% of the HF group and 28% of the CF group (Table 2). Comparing the distribution of maximum late side effects between patients treated with HF or CF, we found significant differences between maximum late gastrointestinal toxicity (*p* = 0.01) and maximum late genitourinary toxicity (*p* = 0.001) in favor of the HF group.

**Table 2:** Maximum late gastrointestinal and genitourinary toxicity after moderate hypofractionation (*HF*) and conventional fractionation (*CF*)

Late Toxicity	Gastrointestinal		Genitourinary	
	HF (%)	CF (%)	HF (%)	CF (%)
*RTOG grade 4*	0.9	0.0	0.0	0.0
*RTOG grade 3*	1.9	2.6	0.0	5.3
*RTOG grade 2*	4.7	13.8	11.3	23.0
*RTOG grade 1*	16.0	21.7	26.4	26.3
*RTOG grade 0*	76.4	61.8	62.3	45.4

The *p*-value when comparing the distribution of maximum late gastrointestinal toxicity between the group treated with HF and CF is *p* = 0.01 and 0.001 when comparing maximum late genitourinary toxicity, respectively. N = 106 for HF and 152 for CF

**Fig. 2:**
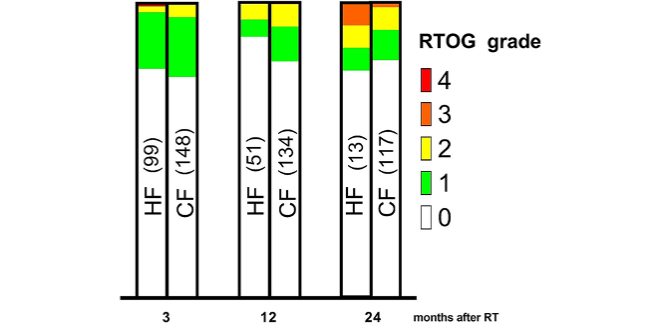
Prevalence of gastrointestinal side effects after treatment over a follow-up period of 24 months. The number of patients is found in brackets. When comparing the distribution of gastrointestinal toxicity for each time point, the *p*-values are *p* = 0.59, 0.22 and 0.71 after 3, 12, and 24 months. *HF* hypofractionation, *CF* conventional fractionation

**Fig. 3:**
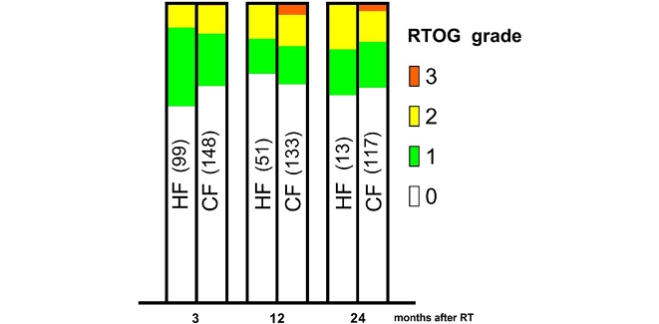
Prevalence of genitourinary side effects after treatment over a follow-up period of 24 months. The number of patients is found in brackets. When comparing the distribution of genitourinary toxicity for each time point, the *p*-values are *p* = 0.39, 0.58 and 0.90 after 3, 12, and 24 months. *HF* hypofractionation, *CF* conventional fractionation

However, there was a large difference in follow-up times between groups. Therefore, we also investigated the distribution of prevalence of late GI and GU toxicities after 3, 12, and 24 months in both groups. These analyses did not show any significant differences between the groups (Figs. 2 and 3). One GI grade‑4 toxicity was reported in a patient treated with HF. This toxicity consisted of an ileus, and it required surgery, but did not require a stoma.

**Fig. 4:**
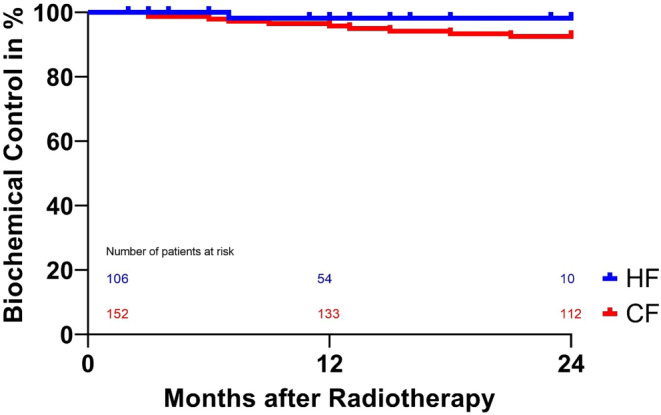
Biochemical control after treatment with either conventional fractionation (*CF*) or moderate hypofractionation (*HF*). *P* = 0.20

Biochemical control rates, as displayed in Fig. 4, are excellent in both groups with 98% in the HF and 93% in the CF group, without any significant difference (*p* = 0.20).

## Discussion

As of now, the question of whether to perform WPRT remains unresolved. The guidelines do not provide a clear recommendation for or against WPRT [15, 16, 25]. Therefore, it is not within the scope of this paper to discuss the pros and cons of WPRT. There is, however, evidence by the POP-RT trial published in 2021, that WPRT in patients with high-risk prostate cancer improves biochemical control and disease-free survival [14], potentially leading to an increased use of WPRT. The reported toxicities from this trial with below 10% RTOG grade 2 late GI toxicity and a tiny bit above 10% RTOG grade 2 late GU toxicity in the WPRT group within the first two years are similar to our reported results [27].

Any new treatment that increases the side effects has to be counterbalanced by an increase in efficacy, or it cannot gain approval. In the CHHiP trial, where 74 Gy delivered at 2 Gy/fraction was compared to 60 Gy delivered at 3 Gy/fraction, the arms did not show any significant differences regarding tumor control [9] or regarding acute [28] and late [9] side effects. Similarly, the present study did not include a dose escalation, but we observed that the HF group did not display increased toxicities compared to the CF group. However, due to our relatively short follow-up, the toxicity results should be interpreted with caution. Nevertheless, the late side effects were similar between groups (Figs. 2 and 3). Thus, overall, moderate hypofractionation did not display any disadvantages related to toxicity, compared to conventional fractionation, within the first two years after treatment.

We were able to identify 3 publications covering the topic of WPRT hypofractionation. The first one is a small retrospective study with only 22 patients, applying WPRT with 2.75 Gy in 15 fractions and a HDR-brachytherapy or stereotactic boost to the prostate, showing acceptable GI and GU toxicity [29]. The other 2 are also mentioned in a recently published meta-analysis [19], covering the topic of moderate hypofractionation for prostate cancer. We summarized both trials, as well as our study, in Table 3. When comparing our study to the others, we remain the only study to our knowledge including a control group. Comparing toxicities between the studies is difficult, as each study uses a different comparison. However, all studies conclude that hypofractionated WPRT is well tolerated.

**Table 3:** Overview of recently published studies

Study	Faria et al.	Maulik et al.	This study
Number of patients	105	120	106 HF/157 CF
Data collection	Prospectively	Retrospectively	Retrospectively
Treatment technique	IMRT	IMRT	IMRT/VMAT
Single dose prostate	3	3	3 HF/2 CF
Single WPRT dose in Gy	2.2	2.2	2.3 HF/1.8–2 CF
Number of fractions	20	20	20 HF/39 CF
Control group	No	No	Yes
Median follow-up in months	74	70	12 HF/57 CF
Toxicity measurement	CTCAE	LENT-SOM	RTOG
ADT prescribed in %	100	100	62.3 HF/81.5 CF
ADT duration in months	18 (Median)	24–36	8.5 HF/15 CF (Median)
bNED	85% after 5 years, 81% after 7 years	93% after 3 years, 80% after 5 years	–
OS	91% after 5 years, 87% after 7 years	–	–
Acute grade 2 or higher GI toxicity	17%	–	46.7% HF/38.9% CF
Acute grade 2 or higher GU toxicity	17%	–	22.9% HF/31.8% CF
Late maximum grade 2 or higher GI toxicity	7%	15%	7.5% HF/16.4% CF
Late maximum grade 2 or higher GU toxicity	9%	19%	11.3% HF/28.3% CF

The recommended dose applied to the pelvis by the NRG guidelines ranges from 44 to 47 Gy in 20 fractions [18]. However, as shown by the CHHiP trial, a dose difference of 3 Gy might lead to a significantly worse outcome [9]. Therefore, the published 44 Gy in 20 fractions to the pelvis [20] might hypothetically lead to a worse tumor control when compared to our 46 Gy.

This study had some limitations. First, the study had the limitations inherent in a retrospective analysis. Second, we included a medium-sized sample and a limited follow-up in the HF group. Besides, also the amount of ADT varies between groups, although Bolla et al. report no relevant differences regarding severe urinary and gastrointestinal toxicity [30]. The difference in prescribed ADT stems most likely from the combination of more patients with intermediate-risk prostate cancer in the HF group, in combination with the NCCN guidelines not recommending ADT for patients with favorable intermediate-risk prostate cancer [15]. However, due to the monocentric character, we reduced the interobserver bias, because all physicians were supervised by the same senior physician during treatment and data collection. Another limitation was the fact that, although the acute side effects were evaluated at the end of treatment, the treatment times differed between the two groups; this difference might have led to underreporting of the toxicities, particularly in the HF group. However, the CHHiP trial showed similar results, based on the peak of acute toxicities in the CF and HF arms, measured after the same treatment times [28]. Finally, the significantly lower maximum late side effects after HF, shown in Table 2, should be interpreted cautiously, because the follow-up times differed between the HF and CF groups; thus, the longer follow-up time might have led to an apparent increase in the maximum toxicity and an underestimation of toxicity in the HF group, especially in the prevalence after 12 and 24 months. For 5 patients in the CF group, late toxicity data was missing. On top of that, missing patient reported outcome measures is also a limitation. In a small analysis, we were able to show that there is no major difference between patients treated with and without GFM regarding toxicity [31], even with increased safety margin without GFM, as long as dose contraints are respected. Therefore, the lack of GFM implantation due to the COVID pandemic might not matter much. However, patients treated with HF reveived fewer GFM implantations is another limitation. On the other hand, since most of the patients in the HF group and most patients of the CF group had 7 mm PTV margins, the margin differences itself are minor. Besides, as the comparison of fractionations is not only between 2 and 2.3 Gy per fraction for the whole pelvis, but also between 2 and 3 Gy per fraction for the prostate, an isolated statement is therefore not pissible. This study can therefore be regarded as a pilot study, displaying that moderate pelvic hypofractionation can be safely used without adding clinically relevant excess toxicity. However, our study is the only one evaluating hypofractionated WPRT while also including a control group.

## Conclusion

With this study, we provided more data on the effects of hypofractionation at a specific dose for WPRT. Our results demonstrated that moderately hypofractionated WPRT could provide safe, efficient treatment, without increasing toxicity, at least in the first years after treatment. In future, longer follow-up times and prospectively randomized controlled trials are needed to confirm these findings.

### Supplementary Information

Appendix 1: Dose constraints for radiation treatments
