# Supplementary material for: Assessing the toxicity after moderately hypofractionated prostate and whole pelvis radiotherapy compared to conventional fractionation
Source: Strahlenther Onkol. 2023 Jun 21;200(3):188–94. doi: 10.1007/s00066-023-02104-7 (PMC10876811; doi:10.1007/s00066-023-02104-7)
Supplement: Supplementary file 1 — Appendix 1: Dose constraints for radiation treatments [file 66_2023_2104_MOESM1_ESM.docx]

Appendix 1 contains the dose constraints for radiation treatments.

Appendix 1: Dose constraints for radiation treatments

| Structure |  | 60 Gy / 46 Gy |  | 78 Gy / 50(.4) Gy |
| --- | --- | --- | --- | --- |
| PTV | D 2% | 107% | D 2% | 107% |
|  | D 50% | Prescribed dose | D 50% | Prescribed dose |
|  | D 95% | 95% | D 95% | 95% |
| Rectum | Dmax | 66 Gy | Dmax | 85.8 Gy |
|  | V 54 Gy | 20% | V 70 Gy (%) | 20% |
|  | V 48 Gy | 40% | V 65 Gy (%) | 40% |
|  | V 40 Gy | 45% | V 60 Gy (%) | 45% |
|  | V 30 Gy | 60% | V 50 Gy (%) | 50% |
| Bladder | Dmax | 66 Gy | Dmax | 85.8 Gy |
|  | V 54 Gy | 20% | V 70 Gy (%) | 20% |
|  | V 48 Gy | 40% | V 55 Gy (%) | 45% |
|  | V 44 Gy | 50% | V 50 Gy (%) | 50% |
|  | V 30 Gy | 80% | V 30 Gy (%) | 80% |
| Bowel Bag | Dmax | 54 Gy | Dmax | 56 Gy |
|  | V 48 Gy | 10% | V 50 Gy (%) | 15% |
|  | V 44 Gy | 15% |  |  |
|  | V 40 Gy | 20% |  |  |
| Femoral head | Dmax | 50 Gy | Dmax | 60 Gy |
|  | V 46 Gy (%) | 5% | V 50 Gy (%) | 5% |

PTV: Planned target volume

Definitions of organs at risk:

rectum = from the anorectal verge to the recto-sigmoidal junction

bladder = whole bladder

bowel bag = “inferiorly, from the most inferior small or large bowel loop or above the rectum […], whichever is most inferior”,^24^ and lateral and ventral to the abdominal wall, up to 2 cm above the PTV
